# Reactions of Bromine Fluoride Dioxide, BrO_2_F, for the Generation of the Mixed‐Valent Bromine Oxygen Cations Br_3_O_4_^+^ and Br_3_O_6_^+^

**DOI:** 10.1002/anie.201912271

**Published:** 2019-11-13

**Authors:** Konrad Seppelt

**Affiliations:** ^1^ Institut für Chemie und Biochemie Freie Universität Berlin Fabeckstrasse 34–36 14195 Berlin Germany

**Keywords:** bromyl fluoride, bromine oxygen compounds, crystal structures, mixed-valent compounds

## Abstract

A reliable synthesis of unstable and highly reactive BrO_2_F is reported. This compound can be converted into BrO_2_^+^SbF_6_^−^, BrO_2_^+^AsF_6_^−^_,_ and BrO_2_^+^AsF_6_^−^⋅2 BrO_2_F. The latter decomposes into mixed‐valent Br_3_O_4_⋅Br_2_^+^AsF_6_^−^ with five‐, three‐, one‐, and zero‐valent bromine. BrO_2_^+^ H(SO_3_CF_3_)_2_^−^ is formed with HSO_3_CF_3_. Excess BrO_2_F yields mixed‐valent Br_3_O_6_^+^OSO_3_CF_3_^−^ with five‐ and three‐valent bromine. Reactions of BrO_2_F and MoF_5_ in SO_2_ClF or CH_2_ClF result in Cl_2_BrO_6_^+^Mo_3_O_3_F_13_^−^. The reaction of BrO_2_F with (CF_3_CO)_2_O and NO_2_ produces O_2_Br‐O‐CO‐CF_3_ and the known NO_2_^+^Br(ONO_2_)_2_^−^. All of these compounds are thermodynamically unstable.

## Graphical abstract

**Thermodynamically unstable**: A reliable synthesis of unstable and highly reactive BrO_2_F is reported. This compound can be converted into BrO_2_^+^AsF_6_^−^⋅2 BrO_2_F, which decomposes into Br_3_O_4_⋅Br_2_^+^AsF_6_^−^ with five‐, three‐, one‐, and zero‐valent bromine. Br_3_O_6_^+^SO_3_CF_3_^−^ contains three‐ and five‐valent bromine.

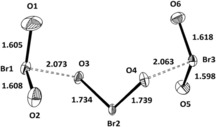

Bromine fluoride dioxide (bromyl fluoride) has long been known,^1^ and its pyramidal structure has been established by spectroscopic methods.^2^ It is a very reactive and unstable species that decomposes above 10 °C, often with explosion. Herein, we present a reliable and safe procedure for its high‐yielding preparation in a PFA tube system between −78° and −10 °C in amounts of 100–200 mg [Eq. (1)].

(1) $$2\;\mathrm{NaBrO}{}_{3}+\mathrm{BrF}{}_{5}+2\;\mathrm{HF}\to 3\;\mathrm{BrO}{}_{2}F+2\;\mathrm{NaHF}{}_{2}$$

A previous single‐crystal determination had suffered from O/F disorder.^3^ However, recrystallization from acetone at low temperatures produced several adducts. In the adduct 3 BrO_2_F⋅4 acetone, the bond lengths are undisturbed by disorder: *r*_BrO_=1.587–1.620(2) and *r*_BrF_=1.781–1.822(2) Å. Solutions in SO_2_ClF or CH_2_ClF are stable at low temperature if all reductive reagents (H_2_O!) are excluded. Even in anhydrous HF slow decomposition occurs (Scheme 1).

**Scheme 1:**
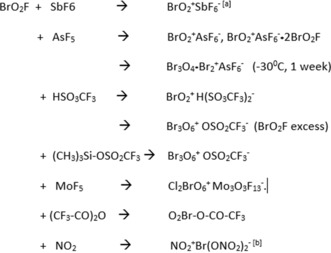
Reactions of BrO_2_F. [a] See Ref. 4. [b] See Ref. 5.

SbF_5_ and BrO_2_F form BrO_2_^+^SbF_6_^−^. This product is identical to the one that has been obtained recently in the reaction of BrO_3_F with SbF_5_ under loss of oxygen.^4^ AsF_5_ works in the same way as SbF_5_, giving BrO_2_^+^AsF_6_^−^. This compound can be sublimed with some decomposition in vacuum at 10 °C. This indicates that the fluoride ion affinity of AsF_5_ is just large enough for the formation of this ionic species. AsF_5_ as a gas can easily be applied in various amounts relative to BrO_2_F: In a reaction with excess BrO_2_F, crystals of BrO_2_^+^AsF_6_^−^⋅2 BrO_2_F are formed. These turned into dark‐red Br_3_O_4_⋅Br_2_^+^AsF_6_^−^ under loss of oxygen after standing for days at −30 °C.

The cation Br_3_O_4_^+^⋅Br_2_ of this salt is shown in Figure 1. The Br_2_ part of the cation can be described as a Br_2_ molecule attached to the Br‐O part of the cation: The Br−Br bond length of 2.280(1) Å), the Br−Br⋅⋅⋅Br bond angle of 104.8(1)°, and the corresponding Raman line of 297.5 cm^−1^ are typical for molecular bromine bonded through halogen bonding. The Br_3_O_4_^+^ cation can be viewed as a combination of BrO_2_^+^ and neutral O=Br‐O‐Br or as O_2_Br‐O‐Br^+^‐O‐Br. In each description, it contains one‐, three‐, and five‐valent bromine (in addition to the zero‐valent Br_2_).

**Figure 1:**
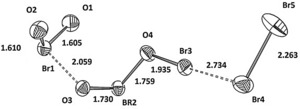
Cation 1 in Br_3_O_4_^+^⋅Br_2_AsF_6_^−^. Cation 2 (almost identical) and anions are omitted. Displacement parameters (also in all figures below) set at 50 %. Distances given in Å. Angles: O1‐Br1‐O2 110.6°, O3‐Br2‐O4 103.5°, O4‐Br3⋅⋅⋅Br4 177.0°.

HSO_3_CF_3_ dissolves BrO_2_F under formation of BrO_2_^+^ H(SO_3_CF_3_)_2_^−^. The anion H(SO_3_CF_3_)_2_^−^ has only occasionally been observed;^6^ the non‐symmetric O−H⋅⋅⋅O bridge here is 2.515 Å long, as compared to 2.410 Å in Ref. 6.

When an excess of BrO_2_F relative to HSO_3_CF_3_ was applied, brown crystals of Br_3_O_6_^+^SO_3_CF_3_^−^ were obtained. The cation of Br_3_O_6_^+^SO_3_CF_3_^−^ can be described as a combination of two BrO_2_^+^ units and one BrO_2_^−^ that weekly interact. The geometries of the two BrO_2_^+^ units are very similar to those observed in the neat BrO_2_^+^ compounds. Little is known about bromite, BrO_2_^−^: The preparation of NaBrO_2_ is quite tedious.^7^ A crystal structure determination on NaBrO_2_⋅3 H_2_O reveals *r*_Br‐O_=1.701(2), 1.731(2) Å, and *δ*_O‐Br‐O_=105.3(1)°.^8^ For our BrO_2_^−^ unit, these data are *r*_Br‐O_=1.733(1), 1.739(1) Å, and *δ*_O‐Br‐O_=102.7(1)°. The Br_3_O_6_^+^ cation is overall close to *C*_2_ symmetry. Aside from the description as BrO_2_^+^⋅BrO_2_^−^⋅BrO_2_^+^, this cation could also be described as a Br^III^–dibromate(V) cation, albeit with two extreme long central bromine–oxygen bonds (Figure 2).

**Figure 2:**
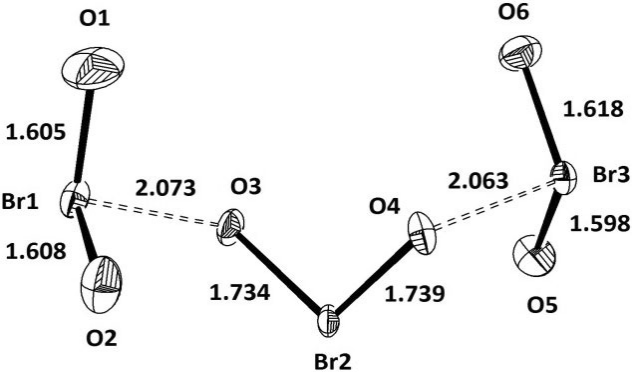
The cation Br_3_O_6_^+^ in Br_3_O_6_^+^ OSO_2_CF_3_^−^; distances in Å. Angles: O1‐Br1‐O2 110.3°, O3‐Br2‐O4 102.8°, O5‐Br3‐O6 108.9°.

BrO_2_F and (CH_3_)_3_Si‐OSO_2_CF_3_ in SO_2_ClF also react to Br_3_O_6_^+^SO_3_CF_3_^−^, now in the form of a yellow fine powder, as confirmed by its identical Raman spectrum (see the Supporting Information).

In speculations about the formation of these mixed‐valent cations, the intermediacy of the free radical ^.^BrO_2_ could be considered. In contrast to long‐known ^.^ClO_2_, it has never been isolated. It has been detected in matrices,^9^ by microwave,^10^ and UV/Vis spectroscopy,^11^ and it has been postulated as a central intermediate in the Belousov–Zhabotinsky oscillating reaction.^12^ We often observed violet solutions in our reactions, although always for only a short period of time. This species seems to dimerize at low temperature, similar to ^.^ClO_2_.^13^ A dimer Br_2_O_4_ might dissociate into BrO_2_^+^BrO_2_^−^, which in turn could react with BrO_2_^+^ to Br_3_O_6_^+^. Obviously not many cases of such a radical dimer dissociation into an ion pair are known; the dissociation of N_2_O_4_ into solid NO^+^NO_3_^−^ in the presence of IF_5_ is one example.^14^

The reaction of BrO_2_F with MoF_5_ in SO_2_ClF or CH_2_ClF offers another surprise: Aside from an ochre‐colored powder and colorless crystals, a red‐brown crop of crystals was always obtained, with the composition Cl_2_BrO_6_^+^Mo_3_O_3_F_13_^−^. The cation can be formulated as ClO_2_^+^⋅BrO_2_^−^⋅ClO_2_^+^, similar to BrO_2_^+^⋅BrO_2_^−^⋅BrO_2_^+^. Because of the extreme oxidation power of BrO_2_F, a lot of atom scrambling has obviously occurred with the solvents (Figure 3).

**Figure 3:**
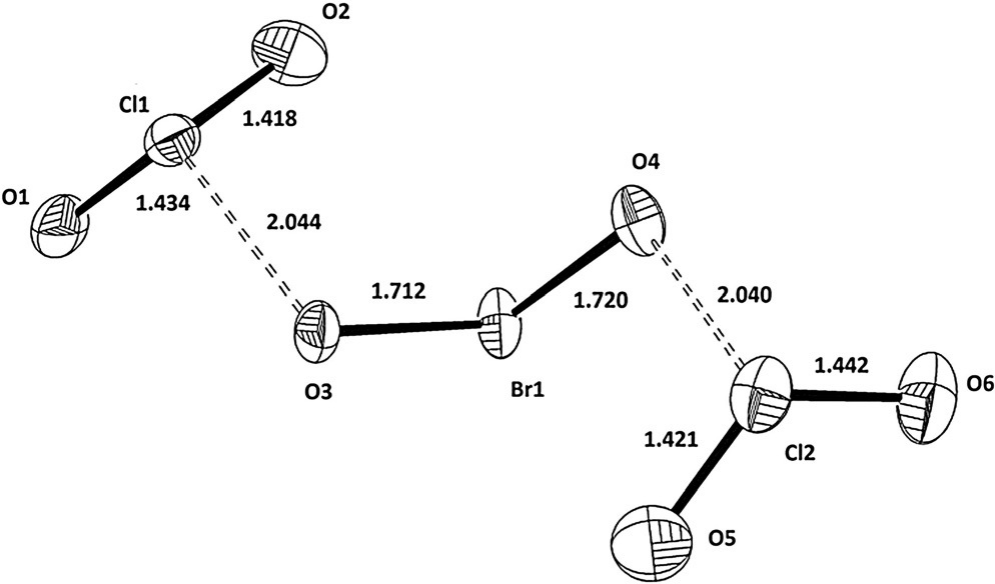
The cation BrCl_2_O_6_^+^ in BrCl_2_O_6_^+^OSO_2_CF_3_^−^; distances in Å. Angles: O1‐Cl1‐O2 116.0°, O3‐Br1‐O4 105.1°, O5‐Cl2‐O6 115.7°.

The reaction of BrO_2_F with neat (CF_3_‐CO)_2_O affords O_2_Br‐O‐CO‐CF_3_ as a pale‐yellow solid that melts at −12 °C, and inevitably explodes upon further warming (Figure 4).

**Figure 4:**
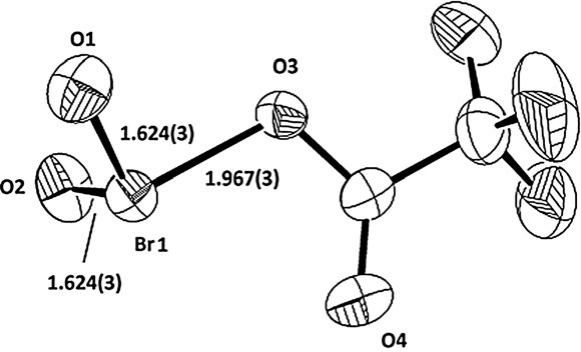
Molecule 1 in the crystal structure of O_2_Br‐O‐CO‐CF_3_; distances in Å. Angles: O1‐Br1‐O2 110.3°, O1‐Br1‐O3 98.5°, O2‐Br1‐O3 97.3°. The three independent molecules in the unit cell differ mainly only in the torsion of the CF_3_ group.

The reaction of BrO_2_F with NO_2_ gives the known compound NO_2_^+^Br(ONO_2_)_2_^−^ in quantitative yield as a colorless crystalline solid, formerly made from N_2_O_5_ and Br‐ONO_3_.^5^ The central Br^I^ is linearly bonded to two oxygen atoms, as expected, and the overall structure is centrosymmetric (Figure 5).

**Figure 5:**
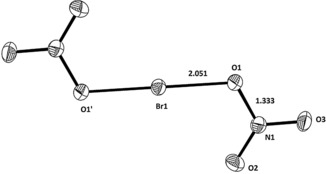
The anion Br(NO_3_)_2_^−^ in NO_2_^+^Br(NO_3_)_2_^−^; distances in Å. Angles: Br1‐O1‐N1 116.2°; sum of angles at N1: 360.0°.

The structures of the cations Br_3_O_4_^+^, Br_3_O_6_^+^, BrCl_2_O_6_^−^, of the compound O_2_Br‐OCO‐CF_3_, and of the anion Br(NO_3_)_2_^−^ have been calculated by the methods B3LYP, MP2, and B97D. Whereas the direct bonds and angles were satisfactorily reproduced, the contact lengths between the units in Br_3_O_4_^+^, Br_3_O_6_^+^, and BrCl_2_O_6_^+^ were too long. The B3LYP method gives the best results among the three methods. However, the long‐distance interactions are still so far off from the experimental values that the calculations of the vibrational spectra are unreliable (see the Supporting Information).

The generation of a thus far non‐reproducible by‐product Cl_2_BrO_6_^+^ ClO_4_^−^ in a reaction of BrO_2_F/HSO_3_CF_3_^−^/SO_2_ClF is reported in the Supporting Information, only to show that more of these compounds can exist. Long ago, a compound described as BrO_2_^+^ClO_4_^−^ was made by ozonization of BrOClO_3_ in CFCl_3,_ but solely characterized by Cl/Br analysis.^15^

## Experimental Section

The generation of BrO_2_F from NaBrO_3_, BrF_5_, and HF is most easily performed on a metal vacuum line in a PFA tube (poly(perfluoroethene perfluorovinyl ether) co‐polymer) at −78 °C, and subsequent sublimation at −10 °C into a second PFA trap cooled to −78 °C. The product obtained is completely colorless. The same reaction without a metal vacuum line is described in detail in the Supporting Information, as are the reactions of BrO_2_F with SbF_5_, AsF_5_, HSO_3_CF_3_, (CH_3_)_3_Si‐OSO_2_CF_3_, MoF_5_, (CF_3_‐CO)_2_O, and NO_2_.

## Conflict of interest

The authors declare no conflict of interest.

## Supporting information

As a service to our authors and readers, this journal provides supporting information supplied by the authors. Such materials are peer reviewed and may be re‐organized for online delivery, but are not copy‐edited or typeset. Technical support issues arising from supporting information (other than missing files) should be addressed to the authors.

Supplementary
