# Supplementary material for: Reactions of Bromine Fluoride Dioxide, BrO2F, for the Generation of the Mixed‐Valent Bromine Oxygen Cations Br3O4 + and Br3O6 +
Source: Angew Chem Int Ed Engl. 2019 Nov 13;58(52):18928–30. doi: 10.1002/anie.201912271 (PMC6973041; doi:10.1002/anie.201912271)
Supplement: Supplementary file 1 — Supplementary [file ANIE-58-18928-s001.pdf]

Angewandte Chemie
Eine Zeitschrift der Gesellschaft Deutscher Chemiker

Supporting Information

Reactions of Bromine Fluoride Dioxide, BrO_2F, for the Generation of the Mixed-Valent Bromine Oxygen Cations $Br_3O_4^+$ and $Br_3O_6^+$

*Konrad Seppelt**

anie_201912271_sm_miscellaneous_information.pdf

Section S1 General, Computational Details, Syntheses, and Raman spectra S2

Table S1 Crystallographic data: : $(BrOF_2)_3$*(acetone)$_4$, O_2Br-O-CO-CF_3 S5

Table S2 Crystallographic data: $BrO_2^+AsF_6^-$, $BrO_2^+AsF_6^-$*$2BrO_2F$,

$Br_3O_4^+$*Br_2 AsF_6^-, BrO_2^+ $H(OSO_2CF_3)_2^-$ S6

Table S3 Crystallographic data: $Br_3O_6^+$ $OSO_2CF_3^-$, $Cl_2BrO_6^+$ $Mo_3O_3F_{13}^-$,

$Cl_2BrO_6^+$ ClO_4^- $HF_{0.5}$, NO_2^+ $Br(ONO_2)_2^-$ S7

Table S4 Selected bond lengths (Å) and angles (deg) of $(BrOF_2)_3$*(acetone)$_4$ S8

Figure S1 Ortep representation: $(BrOF_2)_3$*(acetone)$_4$ S8

Table S5 Selected bond lengths (Å) and angles (deg) of $BrO_2^+AsF_6^-$ S9

Figure S2 Ortep representation: $BrO_2^+AsF_6^-$ S9

Table S6 Selected bond lengths (Å) and angles (deg) of $BrO_2^+AsF_6^-$* $2BrO_2F$ S10

Figure S3 Ortep representation: $BrO_2^+AsF_6^-$* $2BrO_2F$ S10

Table S7 Selected bond lengths (Å) and angles (deg) of $Br_3O_4^+$ *Br_2 AsF_6 S11

Figure S4 Ortep representation: $Br_3O_4^+$ *Br_2 AsF_6^- S11

Table S8 Selected bond lengths (Å) and angles (deg) of BrO_2^+ $H(OSO_2CF_3)_2^-$ S12

Figure S5 Ortep representation: BrO_2^+ $H(OSO_2CF_3)_2^-$ S12

Table S9 Selected bond lengths (Å) and angles (deg) of $Br_3O_6^+$ $OSO_2CF_3^-$ S13

Figure S6 Ortep representation: $Br_3O_6^+$ $OSO_2CF_3^-$ S13

Table S10 Selected bond lengths (Å) and angles (deg) of $Cl_2BrO_6^+$ $Mo_3O_3F_{13}^-$ S14

Figure S7 Ortep representation: $Cl_2BrO_6^+$ $Mo_3O_3F_{13}^-$ S14

Table S11 Selected bond lengths (Å) and angles (deg) of $Cl_2BrO_6^+$ ClO_4^- *$HF_{0.5}$ S15

Figure S8 Ortep representation: $Cl_2BrO_6^+$ ClO_4^- *$HF_{0.5}$ S15

Table S12 Bond lengths (Å) and angles (deg) of O_2Br-O-COCF_3: S16

Figure S9 Ortep representation of O_2Br-O-C-OCF_3. S16

Table S13 Selected bond lengths (Å) and angles (deg) of NO_2^+ $Br(ONO_2)_2^-$ S17

Figure S10 Ortep representation: NO_2^+ $Br(ONO_2)_2^-$ S17

Table S14 Calculated vibrational spectra and structure data S18

References S23

General, Computational Details, Syntheses, and Raman spectra

Materials: $NaBrO_3$, SbF_5, AsF_5, $(CH_3)_3Si\text{-}OSO_2CF_3$, HSO_3CF_3, SO_2ClF, CF_2Cl_2, $(CF_3CO)_2O$, and NO_2 have been on laboratory stock and are used without further purification. BrF_5 usually contains some elemental bromine and can be used as such. Anhydrous HF has been sufficiently dried by double vacuum distillation in a metal vacuum line. Acetone has been dried by shaking with a small amount of P_2O_5 and subsequent vacuum distillation. CH_2ClF has been purchased from ABCR Co, Karlsruhe, Germany.

The vacuum line is made of glass, equipped with a turbo-pump system (vacuum better that 10^{-4} mbar) and a liquid nitrogen cooled trap, filled with soda-lime for absorption of HF etc. The reactions are usually carried out in PFA tubes (poly perfluoroethene-perfluorovinylether co-polimerisate) of 10 mm outer diameter, closed by stainless steel valves.

Instruments: Single Crystal X-ray diffraction data were collected on a Bruker D8 Venture diffractometer with a Photon 100 CMOS area detector, using Mo Kα-radiation. X-ray structures were solved and refined using SHELXL programs.[S1] Raman spectra are taken on a Bruker MultiRam II with a low temperature Ge detector (1064 nm, 30 – 80 mW, resolution 2 cm^{-1}).

Computational methods: Gaussian System from Gaussian Inc. 2019 and 2016, Carnegie Mellon University[S2], and run at the Freie Universität Computer Center ZEDAT. Methods: B3LYP, MP2, and B97D, as implemented in the Gaussian system.

Syntheses:*Warning: Precoutions against fluoride burning and explosions by wearing suitable eye and hand protections have to be taken. The amounts described below should not be increased, since BrO_2F and most of its reaction products will inevitably explode in air or at room temperature. The largest preparations we made was triple as large as the amount described below.*

Bromylfluoride, BrO_2F: A 10 mm outer diameter PFA tube of 40 cm length is bent (without warming) into a W-shaped form and kept as such by fastening it with wire. Into one of the bends e.g. 1 mmole (151 mg) finally powdered $NaBrO_3$ is filled. Both open ends are attached to stainless steel valves. The opposite end to the $NaBrO_3$ filling is attached to the vacuum line and the tube is carefully evacuated to avoid blowing away some of the $NaBrO_3$. The other end of the system is connected to HF and BrF_5 reservoirs. HF and BrF_5 are condensed onto the liquid nitrogen cooled $NaBrO_3$. In case the HF and BrF_5 reservoirs are light enough that the amount taken can be weighed by difference, 100 mg of each are reasonable amounts. In case that weighing is not possible larger amounts of HF and BrF_5 can be applied without changing the yield, except that the following pumping and sublimation steps will take much longer. After disconnecting the HF and BrF_5 reservoirs the reactions mixture is warmed to -78° C. The mixture becomes liquid, and a vigorous reaction sets in. When this is finished all volatiles are pumped off under slow increasing the temperature to -40° C. Depending on the amounts used of HF and BrF_5 this can take hours, until the formed solid is snow white. Then the other bend of the w-shaped reaction tube is cooled to -78° C, while the solid is warmed to -10° C. Sublimation of the BrO_2F sets in, while residual HF (absorbed on $NaHF_2$) is passed away. The temperature is raised to 0° C, until all BrO_2F is sublimed off, which can take one or two hours. With help of a small heat gun the BrO_2F containing part of

the tube is sealed off, on one or both sides, depending what is done afterword. Weighing out the NaHF is easily done and will give 1 mmole, 62 mg. If the amount is higher then the last sublimation has been incomplete, and the white product will also turn yellow in air. Weighing out the BrO_2F usually gives 200 mg (~ 100%) a colorless, solid.

BrO_2F can be dissolved in cold *Freons* like CH_2ClF, $CFCl_3$. It is well soluble also in acetone, acetonitrile, or propionitrile, but then cooling is essential.

Crystal structure of $(BrO_2F)_3$*(acetone)$_4$. We have observed so far three adducts between BrO_2F and acetone: BrO_2F *acetone, $(BrO_2F)_3$ *(acetone)$_4$, $(BrO_2F)_4$ *(acetone)$_5$. Only the crystal structure of $(BrO_2F)_3$ *(acetone)$_4$ had good quality, although the oxygen and fluorine atoms around Br2 seem still to be mutually disorded, at least in part, see table S1.

The following reactions of BrO_2F are usually carried out in the PFA tubes by dissolving 100 – 200 mg freshly prepared BrO_2F in a solvent and adding the reagent, if volatile, by condensation into the PFA tube at liquid N_2 temperature. If the reagent is not easily volatile (SbF_5, HSO_3CF_3, $(CH_3)_3Si$-OSO_2CF_3) at room temperature, then these are added under dry conditions as liquid onto the frozen BrO_2F solution.

$BrO_2^+SbF_6^-$: BrO_2F is dissolved in 1 – 2 ml SO_2ClF at 0° C and cooled to -196° C. SbF_5 in excess is added. After warming a red-orange precipitation has formed. The entire sample is pumped prolonged to dryness, about 2 ml anhydrous HF is condensed into it. Dissolution at room temperature and slow cooling over night to -78° C affords large orange crystals. Crystal data are identical to those as previously published.[S3]

$BrO_2^+AsF_6^-$: BrO_2F is dissolved in 1 – 2 ml SO_2ClF at 0° C and cooled to -196° C. AsF_5 is added in excess by condensation in vacuum at -196° C. Warming to 0° C temperature and slow cooling over night to -78°C affords an orange powder. Puming off the solvent and adding 1.5 ml HF result in orange crystals. Raman spectrum (cm $^{-1}$, assignment, rel. intensity): 931 (BrO_2^+, 30), 864 (BrO_2^+, 100), 687 (AsF_6^-, 80), 556 (AsF_6^-, 35), 364, 349 (BrO_2^+, AsF_6^-, 70, 30). Crystal data see table S2.

$BrO_2^+AsF_6^-$ *BrO_2F: 210 mg (1.4 mmol) BrO_2F are dissolved in 2.5 ml CH_2ClF, and 240 mg (1.4 mmol) are condensed onto it at -196°C. Warming to 0° C temperature and slow cooling over night to 78°C affords red, irregular crystals. Crystal data see table S2.

$Br_3O_4^+$ *Br_2 AsF_6^- : The $BrO_2^+AsF_6^-$ *BrO_2F sample in CH_2ClF is kept for prolonged time at -30° C, yellow, cubic shaped crystals are formed. Raman spectrum (cm $^{-1}$, tentative assignments, rel. intensity): 941 (BrO_2^+,2), 891 (BrO_2^+, 4), 679 (AsF_6^-, 5) , 594 (3), 557(AsF_6^-, 1) , 353 (BrO_2^+, AsF_6^-, 8) , 329 (4) , 298 (Br_2, 100), 261 (7). Crystal data see table S2.

BrO_2^+ $H(OSO_2CF_3)_2^-$: 100 mg BrO_2F is dissolved in 3 ml SO_2ClF, and an excess 400 mg HSO_3CF_3 is pipetted on the to -196°C frozen solution. Upon warming a yellow solid is formed, that upon crystallization from 0°C to -78°C gives a large crop of BrO_2^+ $H(OSO_2CF_3)_2^-$ in form of orange cubes. Crystal data see table S2.

$Br_3O_6^+$ $OSO_2CF_3^-$: 150 mg (1 mmol) BrO_2F is dissolved in 3 ml SO_2ClF, and 100 mg (66 mol) HSO_3CF_3 is pipetted on the to -196°C frozen solution. Upon warming a yellow solid is formed, that upon crystallization from 0°C to -78°C gives a large crop of $Br_3O_6^+$ $SO_3CF_3^-$ in form of yellow cubes. Raman spectrum, (cm $^{-1}$, tentative assignment, rel. intensity): 1343 (2), 1256 (SO_3, 5), 1230 (CF_3, 4), 1206 (4), 1175 (CF_3, 5), 1015 (SO_3, 30), 938 (BrO_2^+, 11), 887 (BrO_2^+, 25), 868 (24), 744 (CF_3, 10), 675 (51), 644 (SO_3, 12), 587 (CF_3, 5), 377 (BrO_2^+, 75), 364 (100), 292 (15), 266 (80), 230 (14), 188 (11). Crystal data see table S3. **$Br_3O_6^+$ $OSO_2CF_3^-$** is also obtained by reacting 180 mg (1.2 mmol) BrO_2F, dissolved in 2.0 ml CH_2ClF, and by adding 270 mg $(H_3C)_3SiO\text{-}SO_2CF_3$ to it at -196°C. After warming to -78°C a vigorous reaction sets under formation of a fine yellow powder. The Raman spectrum is identical to the crystalline product described above.

$Cl_2BrO_6^+$ $Mo_3O_3F_{13}^-$: 300 mg (2 mmol) BrO_2F is dissolved in 3 ml CH_2ClF, and 300 mg (1.65 mmol) MoF_5 are added on to the frozen (-196° C) solution. Upon warming a light-brown precipitate is formed, dissolves as a brown-yellow solution after further warming. Some gas evolves (O_2?). Upon cooling three types of crystals are formed: colorless plates (H_3O^+ $Mo_2O_2F_9^-$), orange needles: $BrCl_2O_6^+$ $Mo_2O_2F_9^-$, and brown platelets: $BrCl_2O_6^+$ $Mo_3O_3F_{13}^-$. The crystal data of $BrCl_2O_6^+$ $Mo_2O_2F_9^-$ and H_3O^+ $Mo_2O_2F_9^-$ are of low quality, and no details of the structures are reported here. Crystal data of $BrCl_2O_6^+$ $Mo_3O_3F_{13}^-$ see table S3.

$Cl_2BrO_6^+$ ClO_4^- : In a reaction between BrO_2F and HSO_3CF_3 in SO_2ClF after long standing at -40° C orange platelets appeared, that according to the single crystal structure determination consist of $Cl_2BrO_6^+$ $ClO_4^-{*}HF_{0.5}$. Raman spectrum (cm $^{-1}$, assignments under the assumption of no interactions between ClO_2^+ and BrO_2^- units, rel. intensity): 1505 (4), 1383 (4), 1233 (ClO_2^+?, 10), 1042 (ClO_2^+, 50), 931 (ClO_4^-, 42), 879 (11), 736 (13), 695 (BrO_2^-, 42), 671 (BrO_2^-, 6), 466 (ClO_4^-, 19), 413 (25), 391 (BrO_2^-, 30), 374 (35), 351 (100), 293 (40), 247 (51), 234 (48). Crystal data of $Cl_2BrO_6^+$ $ClO_4^-{*}HF_{0.5}$ see table S3.

$O_2BrO\text{-}COCF_3$: At -196° C an excess $(CF_3CO)_2O$ is condensed in vacuum on BrO_2F. BrO_2F remains solid and undissolved at -78° C. Slow warming to -30° C result in a vigorous reaction with gas evolution (CF_3COF) and a clear, light yellow solution. Pumping off all volatiles at -78° C and redissolving and recrystallization in $CFCl_3$ affords a large crop of light yellow crystals, mp -12° C. The molten red liquid starts to gas slowly and explodes heavily at room temperature. ^{19}F-nmr (CH_2ClF): -72.44 ppm at -60° C. ^{13}C-nmr (CH_2ClF): 113.2 ppm (CF_3, $^1J_{CF}$ = 284.6 Hz), 160.1 ppm (CO, $^2J_{CF}$ = 40.7 Hz). Mass spectrum (-30° C, 5x10^{-5} mbar) m/e = 192/194 Br-OCO-CF_3^+ and smaller ions. Raman spectrum (cm $^{-1}$, assignment, rel. intensity: 1748 (C=O, 8), 1334, 1324 (C-F, 23), 1147 (C-F, 12), 940 (Br=O, 15), 895 (Br=O, 95), 863 (26), 735 (1), 599 (20), 497 (100), 390, 381 (7), 331 (22), 298 (6), 262 (7), 239, 227 (8), 180 (85). Many of the Raman bands (especially νC=O) seem to be split into two or three components, possibly due to the existence of three crystallographically different molecules in the solid. Crystal data of $O_2BrO\text{-}COCF_3$ see table S1.

NO_2^+ $Br(ONO_2)_2^-$: 150 mg (1 mmol) BrO_2F are dissolved in CF_2Cl_2, and an excess of NO_2 is added at -130°C. Gas evolution (O_2) sets in around -78° C. Keeping the mixture for days at -78° C affords a large crop of colorless, cubic shaped crystals. Raman spectrum (cm $^{-1}$, assignment, rel. intensity): 2286 (NO_2^+, 10), 1528 (NO_2, 30), 1407 (NO_2^+, 45), 1282 (NO_2, 32), 1100 (7), 964 (25), 910 (26), 746 (BrO_2, 100), 697 (6), 507 (8), 314 (61), 179 28), 151 (38). Crystal data of NO_2^+ $Br(ONO_2)_2^-$ see table S3.

Table S1. Crystallographic Data[S4]

	$(BrOF_2)_{3*}(acetone)_4$	$O_2Br\text{-}O\text{-}CO\text{-}CF_3$
formula	$Br_3C_{12}F_3H_{24}O_{10}$	$BrC_2F_3O_6$
formula weight	625.04	224.93
space group	P2(1)/c	Pbca
T (K)	100.03	143(2)
a (Å)	13.1240(11)	13.159(9)
b (Å)	20.9427(14)	9.812(8)
c (Å)	8.46997)	27.28(2)
α (deg)	90.	90.
β (deg)	92. 202(3)	90.
γ (deg)	90.	90.
V (A^3)	2326.25	3522(5)
D_c (g/cm^{-3})	1.785	2.545
μ (mm^{-1})	5.27	7.024
Goodness of fit	1.033	1.064
R_1, wR_2 [I>2s(I)][a]	0.0262,	0.0383, 0.0861
R_1, wR_2 (all data)[b]	0.0338, 0.0533	0.0571, 0.0932

[a] $R_1 = \Sigma||F_o| - |F_c||/\Sigma|F_o|$

[b] $wR_2 = \{\Sigma w[(F_o)^2 - (Fc)^2]^2/\Sigma w[(F_o)^2]^2\}^{1/2}$

Table S2. Crystallographic Data[S4]

	$BrO_2^+AsF_6^-$	$BrO_2^+AsF_6^- * 2BrO_2F$	$Br_3O_4^+ * Br_2\ AsF_6^-$	$BrO_2^+\ H(OSO_2CF_3)_2^-$
formula	$AsBrF_6O_2$	$AsBr_2F_7O_4$	$AsBr_5F_6O_4$	$BrC_3F_6HO_6S$
formula weight	300.83	556.65	652.47	411.06
space group	Cmca	C2/c	P-1	P-1
T (K)	100.01	100.01	100.01	100.03
a (Å)	10.225(2)	15.2236(14)	9.4552(6)	8.5286(5)
b (Å)	11.352(2)	5.7984(5)	9.8778(7)	8.6547(5)
c (Å)	10.157(1)	13.9951(14)	13.1336(9)	8.7542(6)
α (deg)	90.	90.	79.825(2)	106.405(2)
β (deg)	90.	117.961(3)	82.998(2)	93.948(2)
γ (deg)	90.	90.	88.825(2)	113.969(2)
V (A^3)	1178.96	1091.17	1198.34	553.85
D_c (g/cm^{-3})	3.390	3.388	3.617	2.465
μ (mm^{-1})	12.62	14.22	19.58	4.21
Goodness of fit	1.131	1.182	1.066	1.129
R_1, wR_2 [I>2s(I)][a]	0.0490, 0.125	0.0560, 0.1414	0.0207, 0.0446	0.0184, 0.0460
R_1, wR_2 (all data)[b]	0.0548, 0.1287	0.0569, 0.1422	0.0254, 0.0459	0.0204, 0.0470

[a] $R_1 = \Sigma||F_o| - |F_c||/\Sigma|F_o|$

[b] $wR_2 = \{\Sigma w[(F_o)^2 - (Fc)^2]^2/\Sigma w[(F_o)^2]^2\}^{1/2}$

Table S3. Crystallographic Data[S4]

	$Br_3O_6^+$ $OSO_2CF_3^-$	$Cl_2BrO_6^+$ $Mo_3O_3F_{13}^-$	$Cl_2BrO_6^+$ ClO_4^- $HF_{0.5}$	NO_2^+ $Br(ONO_2)_2^-$
formula	$Br_3CF_3O_9S$	$BrCl_2F_{13}Mo_3O_3$	$Cl_3BrF_{0.5}H_{0.5}O_{10}$	BrN_3O_8
formula weight	484.80	829.36	311.31	249.94
space group	C2/c	P-1	Fdd2[c]	$P2_1/n$
T (K)	100.04	100.01	100.13	100.00
a (Å)	19.0494(10)	8.8210(7)	15.7681(15)	6.1105(6)
b (Å)	9.9253(5)	9.7703(8)	19.0587 (18)	6.3991(7)
c (Å)	13.0221(6)	12.3052(10)	12.5509(10)	8.7722(9)
α (deg)	90.	69.179(3)	90.	90.
β (deg)	115.170(2)	86.088(3)	90.	109.980(4)
γ (deg)	90.	63.758(3)	90.	90.
V (A^3)	2228.32	884.07	3771.79	322.36
D_c (g/cm^{-3})	2.890	3.117	2.193	2.575
μ (mm^{-1})	11.13	4.12	4.96	6.40
Goodness of fit	1. 084	1.148	1.077	1.046
R_1, wR_2 [I>2s(I)][a]	0.0135, 0.0308	0.0355, 0. 0605	0.0246, 0.0600	0.0336, 0.0792
R_1, wR_2 (all data)[b]	0.05150, 0.0312	0.0456, 0.0631	0.0259, 0.0606	0.0469, 0.0845

[a] $R_1 = \Sigma||F_o| - |F_c||/\Sigma|F_o|$

[b] $wR_2 = \{\Sigma w[(F_o)^2 - (Fc)^2]^2/\Sigma w[(F_o)^2]^2\}^{1/2}$

[c] Flack factor: 0.0651

Table S4. Bond lengths (Å) and angles (deg) in $(BrO_2F)_{3*}(acetone)_4$:

Br1-O 1.606(2), 1.620(2), Br1···O11 2.662(3), O-Br1-O 107.4(1), O-Br1-F1 99.8(1), 100.3(1).

Br2-O 1.622(2), 1.671(2), Br2-F2 1.7037(2), Br2···O14 2.683(3), O-Br2-O 104.2(1), O-Br2-F2 102.5(1), 99.4(1).

Br3-O 1.587(2), 1.594(2), Br3-F1 1.822(2), Br3···O12 2.562(3), O-Br3-O 107.4(1), O-Br3-F1 99.8(1), 100.3(1).

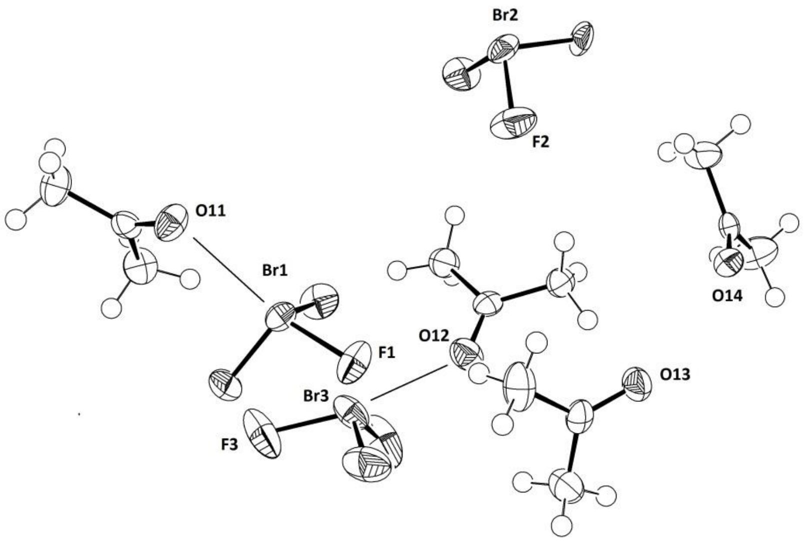

Figure S1: Ortep representation of $(BrO_2F)_{3*}(acetone)_4$, thin lines indicate two of the eight short intermolecluar bromine-acetone interactions.

Table S5. Bond lengths (Å) and angles (deg) of $BrO_2^+AsF_6^-$:

Br-O 1.590(3), O-Br-O 112.1(3),

As-F 1.700 – 1.723(4), F-As-F 89.4(1) – 90.1(1), 178.3(1) – 179.6(1).

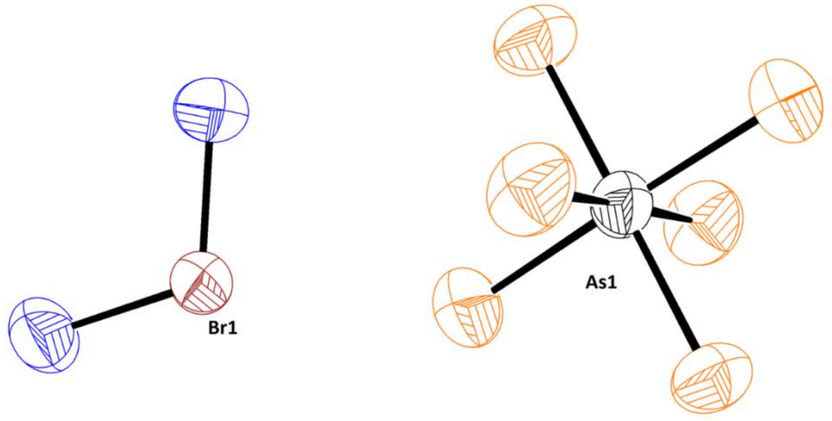

Figure S2: Ortep representation of $BrO_2^+AsF_6^-$

Table S6. Bond lengths (Å) and angles (deg) of $BrO_2^+AsF_6^- * 2BrO_2F$:

(There is obviously a partial disorder among the O and F atoms in the BrO_2F part of the structure.)

BrO_2^+ : Br – O 1.599(4), O-Br-O 111.5(3), BrO_2F: Br-O 1.621(3), 1.672(4), Br-F 1.689(4), O-Br-O 105.2(2), F-Br-O 102.2(2), 104.6(2).

AsF_6^- 1.717(3), 1.731(3), F-As-F 89.5(2) – 90.4(2), 180.

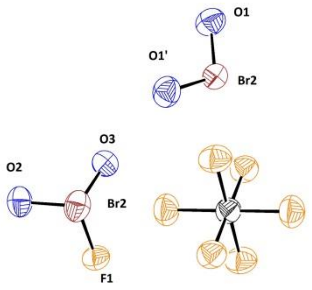

Figure S3: Ortep representation of $BrO_2^+AsF_6^- * 2BrO_2F$:

Table S7. Bond lengths (Å) and angles (deg) of $Br_3O_4^{+}{*}Br_2$ AsF_6^{-} :

Cation 1:

Br1-O1 1.610(3), Br1-O2 1.605(3), Br1…O3 2.059(2), Br2-O3 1.730(2), Br2-O4 1.759(2), Br3-O4 1.935(2), Br3…Br4 2.7377(5), Br4-Br5 2.2625(6).

O1-Br1-O2 110.56(1), O3…Br-O1 98.1(1), O3…Br1-O2 96.7(1), Br1…O3-Br2 111.06, O3-Br2-O4 103.5(1), Br2-O4-Br3 115.7(1), O4-Br3…Br4 177.00(8), Br3…Br4-Br5 98.06(2).

Cation 2:

Br6-O5 1.606(3), Br6-O6 1.606(2), Br6…O7 2.083(2), Br7-O7 1.728(2), Br7-O8 1.765(2), Br8-O8 1.919(2), Br8…Br9 2.8205(5), Br9-Br10 2.2805(6).

O5-Br6-O6 109.9(1), O7…Br6-O5 100.3(1), O7…Br6-O6 98.5(1), Br6…O7-Br7 111.8(1), O7-Br7-O8 103.0(1), Br7-O8-Br8 114.1(1), O8-Br8…Br9 177.93(8), Br8…Br9-Br10 104.83(3).

Anions 1 and2:

As1-F 1.795(2)-1.727(2), As2-F 1.705(2)-1.733(2),

F-As1-F 88.9(1)-91.7(1), 178.2(1)-179.7(1), F-As2-F 88.3(1), 177.1(1)-179.5(1).

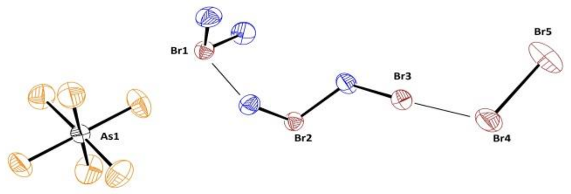

Figure S4: Ortep representation of unit 1, **(**there are two crystallographically different cations and anions in the cell).

Table S8. Bond lengths (Å) and angles (deg) of $BrO_2^+ H(OSO_2CF_3)_2^-$:

Cation:

Br-O 1.5917(16), 1.5933(16), O-Br-O 110.99 (9)

Anion:

S1-O 1.4200(16), 1.4273(16), 1.514(16) S1-C1 1.834(2)

O-S1-O 108.35(1)-120.5(1), C-S2-O 102.8(1)-106.7(1)

S2-O 1.4273(16), 1.4527(16), 1.4717(15), S2-C2 1.832(2)

O-S2-O 111.5(1)- 116.9(1), C-S2-O 103.1(1)-105.8(1)

C1-F 1.319(2)-1.322(2), F-C1-F 108.9(2)-109-4(2), S1-C1-F 109.4(2)-110.1(2)

C2-F 1.318(3)-1.323(3), F-C2-F 108.4(2)-109-3(2), S2-C2-F 109.7(2)-110.7(2)

O5-H1 0.705, O6…H1 1.811, O5…O6 2.512

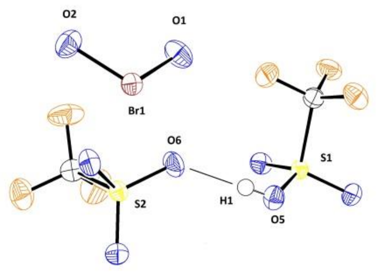

Figure S5: Ortep representation of $BrO_2^+ H(OSO_2CF_3)_2^-$

Table S9. Bond lengths (Å) and angles (deg) of $Br_3O_6^+$ $OSO_2CF_3^-$:

Cation:

Br1-O1,2 1.605(1), 1.608(1), Br1…O3 2.07(1), Br2-O3,4 1.734(1), 1.739(1), Br3…O4 2.063(1), Br3-O5,6 1.598(2), 1.618(2), Br3….O4 2.063(1)

O1-Br1-O2 110.34(8), O1-Br1…O3 97.26(6), O2-Br1…O3 100.59(7), O3-Br2-O4 102.76(7), O5-Br3-O6 108.88(9), O5-Br3…O4 101.85(7), O6-Br3…O4 98.06(7), Br1…O3-Br2 110.14(7), Br2-O4…Br3 112.27(7)

Anion:

S1-O 1.4407(15), 1.4548(14), 1.4604(14) S1-C1 1.8315(22),

O-S1-O 112.91(8)-115.04(8), C-S-O 103.78(9)-104.33(10)

C1-F 1.318(3), 1.320(3), 1.329(3), F-C1-F 108.6(2)-109,5(2), S1-C1-F 108.9(2)-110.2(2)

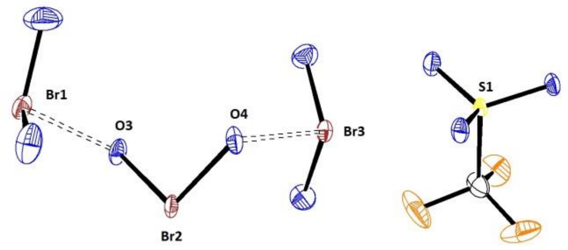

Figure S6: Ortep representation of $Br_3O_6^+$ $OSO_2CF_3^-$**:**

Table S10. Bond lengths (Å) and angles (deg) of $Cl_2BrO_6^+ Mo_3O_3F_{13}^-$

Cation:

Cl1-O1 1.417(2), Cl1-O2 1.435(2), Cl1…O3 2.044(2) Br1-O3 1.712(2), Br1-O4 1.720(2), Cl2-O5 1.442(2), Cl2-O6 1.421(2), Cl2…O4 2.040(2)

O1-Cl1-O2 116.9(2), O3…Cl-O1 100.2(1), O3…Cl1-O2 99.8(1), Cl1…O3-Br1 110.7(1), O3-Br-O4 104.1(1), Br1-O4…Cl2 108.95(1), O4…Cl2-O5 99.5(1), O4…Cl2-O6 101.4(1), O5-Cl2-O6 115.7(2)

Anion: (Slightly distorted octahedral co-ordination round Mo) Mo-O 1.648(2) – 1.668(2), Mo-F (opposite to fluorine atoms) 1.828(2) – 1.869(2), Mo-F (bridging) 2.148 – 2.249(2)

O-Mo-F 94.14(9) – 98.11(10), 176.94(9) – 179.29(10), F-Mo-F 79.25(8) – 92.82(2), 158.9 – 167.6(2)

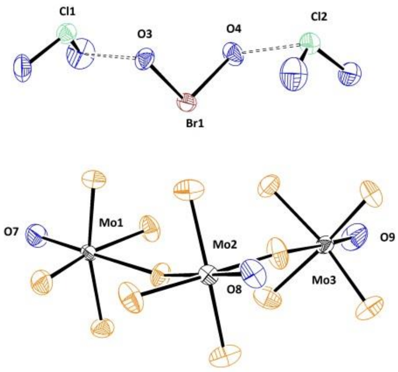

Figure S7: Ortep representation of $Cl_2BrO_6^+ Mo_3O_3F_{13}^-$:

Table S11. Bond lengths (Å) and angles (deg) of $Cl_2BrO_6^+ ClO_4^- HF_{0.5}$**:**

Cation:

Cl1-O1 1.441(5), Cl1-O2 1.443(5), Cl1…O3 2.019(5) Br1-O3 1.726(5), Br1-O4 1.731(4), Cl2-O5 1.452(5), Cl2-O6 1.457(5), Cl2…O4 2.0185)

O1-Cl1-O2 115.5(3), O3…Cl-O1 98.5(3), O3…Cl1-O2 99.8(2), Cl1…O3-Br1 109.8(2), O3-Br-O4 104.2(2), Br1-O4…Cl2 111.7(2), O4…Cl2-O5 98.4(2), O4…Cl2-O6 99.9(2), O5-Cl2-O6 115(3)

Anion:

Cl-O 1.443(4 – 1.454(4), O-Cl-O 109.02(3) – 110.2(3)

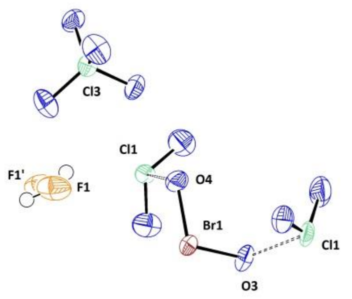

Figure S8: Ortep representation of $Cl_2BrO_6^+ ClO_4^- HF_{0.5}$ **:**

Table S12 Bond lengths (Å) and angles (deg) of O_2Br-O-$COCF_3$:

Molecule 1:

Br=O 1.603(3), 1.624(3), Br-O 1.967(3), C-O 1.336(5), C=O 1.195(5) C-C 1.540(7), C-F 1.317(6), 1.329(6), 1.339(6);

O=Br=O 110.3(2), O=Br-O 98.5(1), 97.3(1), Br-O-C 111.2(3), O-C-C 110.6(4)

Br1-O3-C1-O4 2.4 (5).

Molcule 2 :

Br=O 1.600(3), 1.616(3), Br-O 1.970(3), C-O 1.336(5), C=O 1.203(5) C-C 1.551(7), C-F 1.304(6), 1.321(6), 1.323(6);

O=Br=O 108.5(2), O=Br-O 98.1(2), 102.6(2), Br-O-C 108.9(3), O-C-C 110.3(4)

Br2-O7-C3-O8 1.8(6).

Molecule 3:

Br=O 1.600(3), 1.602(3), Br-O 1.970(3), C-O 1.336(5), C=O 1.195(5) C-C 1.540(7), C-F 1.317(6), 1.329(6), 1.339(6);

O=Br=O 107.9(2), O=Br-O 98.2(2), 98.5(2), Br-O-C 110.8(3), O-C-C 110.3(4)

Br1-O3-C1-O4 3.8 (6).

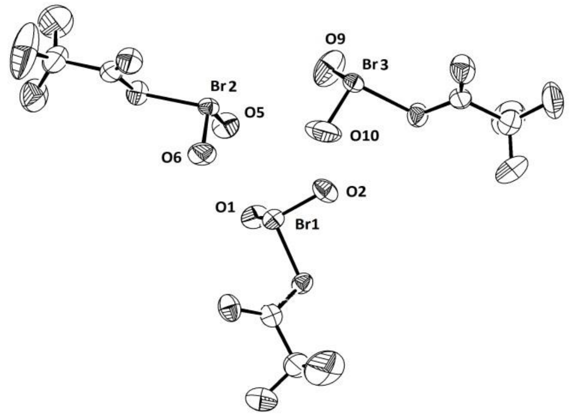

Figure S9: Ortep representation of O_2Br-O-C-OCF_3.

Not shown in figure S9: Eight intermolecular contacts with Br…O < 3.0 Å

Table S13. Bond lengths (Å) and angles (deg) of $NO_2^+ Br(ONO_2)_2^-$:

Cation:

N2-O4 1.115(1), O-N-O 180.0

Anion:

Br1-O1 2.051(1), N1-O1 1.332(2), N1-O2 1.221(2), N1-O3 1.2301(2)

O1-Br1-O1 180.0, Br1-O1-N1 116.2(1), O1-N1-O2 120.3(1), O1-N1-O3 114.4(1), O2-N1-O3 125.4(1)

ΣO-N-O = 360.1

Torsion angles: N1-O1….O2-N2 180, nano Br1-O1-N1-O2: 5.9(2), Br1-O1-N1-O3 -174.6(1).

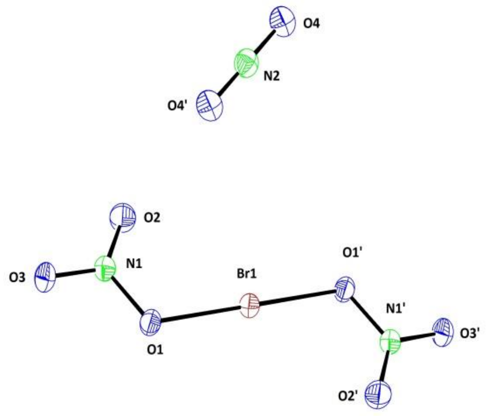

Figure S10: Ortep representation of $NO_2^+ Br(ONO_2)_2^-$:

Table S14 Calculated structure data: bond lengths (Å) and angles (deg), B3LYP, MP2, B89D[S2], basis sets: O, F, N: aug-cc-pVTZ, Br: (10s11p9d1f)/[5s4p3d1f, ECP10MDF).[S5]

$Br_3O_4^+$ (numbering as in Tab. S7)

	B3LYP	MP2	B97D	*X-ray(av., cation 1)*
Br(1)-O	1.611, 1.602	1.665, 1.609	1.634, 1.622	*1.605, 1.610*
Br(1)…O	2.212	2.475	2.349	*2.059*
Br(2)-O	1.711, 1.801	1.646, 1.780	1.710, 1.875	*1.730, 1.759*
Br(3)-O	1.864	1.860	1.852	*1.935*
O-Br(1)-O	113.7	114.0	114.8	*110.6*
O-Br(1)…O	95.3, 101.7	82.6, 98.9	95.0, 101.5	*96.7, 98.1*
Br(1)…O-Br2	113.3	96.9	113.4	*111.8*
O-Br(2)-O	107.4	108.3	109.9	*103.5*
Br(2)-O-Br(3)	117.9	113.3	119.7	*115.7*
E(a.u.)	-1551.078549	-1547.0410567	-1553.4944614	

Calculated vibrational spectra (B3LYP), symmetry C1: in cm^{-1} (rel. Raman intensity, assignment):

989 (15, $\nu_{as}BrO_2^+$), 917 (62, $\nu_sBrO_2^+$), 721 (31, νBr=O), 562 (38, ν_sOBrO), 519 (16, ν_{as}OBrO), 369 (4.3, $\delta_sBrO_2^+$), 338 (31), 291 (3.5), 232 (26), and 6 vibration below 200 cm^{-1}.

$Br_3O_6^+$ (numbering as in Tab. S9)

	B3LYP	MP2	B97D	*X-ray (av.)*
Br(1,3)-O	1.613, 1.603	1.663, 1.610	1.623, 1.632	*1.603*
Br(1,3)…O	2.186	2.450	2.326	*2.042*
Br(2)-O	1.721	1.653	1.718	*1.736*
O-Br(1,3)-O	113.6	113.8	114.8	*109.5*
O-Br(1,3)…O	96.2, 104.5	82.5, 102.3	95.8, 100.4	*97.6, 101.2*
Br(1.3)…O-Br(2)	113.4	97.0	113.6	*111.2*
O-Br(2)-O	107.3	110.7	110.0	*102.8*
E(a.u.)	-1701.419867	-1697.1595548	-1703.7779744	

Calculated vibrational spectra (B3LYP), symmetry C2, in cm^{-1} (rel. Raman intensity, symmetry, assignment):

987 (67, A, $\nu_{as}BrO_2^+$), 986 (8, B, $\nu_{as}BrO_2^+$), 917 (170, A, $\nu_sBrO_2^+$), 912 (170, B, $\nu_sBrO_2^+$), 699 (460, A, $\nu_sBrO_2^-$), 695 (2, B, $\nu_{as}BrO_2^-$), 374 (3, B), 369 (9, A, $\delta_sBrO_2^+$), 343 (1, B), 342 (176, A $\delta_sBrO_2^-$), 313 (31, A), 253 (102, A), 244 (9, B) and 8 vibration below 200 cm^{-1}.

$BrCl_2O_6^+$ (numbering as in Tab. S10)

	B3LYP	MP2	B97D	*X-ray (av.)*
Cl(1,2)-O	1.454, 1.444	1.482, 1.460	1.474, 1.464	*1.418, 1.435*
Cl(1,2)…O	2.152	2.241	2.313	*2.044*
Br-O	1.710	1.679	1.703	*1.716*
O-Cl(1,2)-O	117.0	117.5	117.9	*115.9*
O-Cl(1,2)…O	98.6, 100.1	92.3, 101.4	98.1, 100.4	*99.6, 100,7*
Cl(1,2)…O-Br	112.8	102.0	113.5	*109.8*
O-Br-O	108.1	108.9	111.00	*105.1*
E(a.u.)	-1788.0136672	-1785.0742561	-1788.7065817	

Calculated vibrational spectra (B3LYP), symmetry C2: in cm^{-1} (rel. Raman intensity, symmetry, assignment):

1233 (3.6, B, $\nu_{as}ClO_2^+$), 1233 (57, A, $\nu_{as}ClO_2^+$), 1042 (240, A, $\nu_sClO_2^+$), 1032 (1.2, B, $\nu_sClO_2^+$), 721 (5.5, B, $\nu_sBrO_2^-$), 709 (494, A, $\nu_sBrO_2^-$), 510 (0.4, B, δ_sClO_2), 509 (4.0, A, δ_sClO_2), 374 (0.5, B), 355 (318, A), 345 (4.7, A), 322 (13, B), 318 (127, A), 204 (2.7, B) and 7 vibration below 200 cm^{-1}.

O_2Br-O-CO-CF_3, Cs-symmetry

	B3LYP	MP2	B97D	*X-ray (av.)*
Br=O, Br-O	1.611,2.007	1.591, 2.044,	1.623, 2.105	*1.602, 1.973*
C-O, C=O	1.333, 1.192	1.333, 1.206	1.326, 1.202	*1.315, 1.201*
C-C	1.566	1.591	1.595	*1.547*
C-F	1.328, 1.342	1.325, 1.337	1.338, 1.351	*1.315, 1.330*
O=Br=O	112.57	113.04	1.....0346	*108.9*
O=Br-O	105.51	104.82	106.22	*98.4*
Br-O-C	118.06	112.00	117.3	*110.3*
E(a.u.)	-1093.6025275	- 1091.314232	-1094.1065547	

Calculated vibrational spectra (B3LYP), symmetry Cs: in cm^{-1} (rel. Raman intensity, assignment): 1819 (12, vC=O), 1265 (136, vC-F), 1205 (53, vCF), 1158 (2, vCF), 1109 (vC-O), 969 (12, vBr=O), 915 (41, vBr=O), 842 (25), 772 (0), 729 (1), 590 (23), 516 (1), 485 (36), 402 (1), 365 (3), 306 (7), 282 (1), 280 (29), and 6 vibrations below 200 cm^{-1}.

$Br(ONO_2)_2^-$

	B3LYP	MP2	B97D	*X-ray*
Br-O	2.086	2.032	2.132	*2.051*
O-N	1.333	1.330	1.351	*1.333*
N-O	1.221, 1.227	1.227, 1.233	1.225, 1.234	*1.221, 1.230*
O-Br-O	180.	180.	180.	*180.*
Br-O-N	118.9	116.8	119.6	*116.2*
O-N-O	114.9, 119.9, 125,2	115.0, 116.9, 125.3	113.9, 120.4, 125.7	*114,4, 120.3, 125.4*
E(a.u.)	-977.8089285	-975.6423861	-978.3604417	

Calculated vibrational spectra (B3LYP), overall symmetry C2h: in cm^{-1} (rel. Raman intensity, symmetry, assignment): 1548 (112, Ag, $\nu_{as}NO_2$), 1547 (0.0, Bu, $\nu_{as}NO_2$), 1312 (45, Ag, ν_sNO_2), 1274 (0.0, Bu, ν_sNO_2), 985 (20, Ag, ν_sNO) 953 (0.0 Bu, ν_sNO), 806 (0.1, Bg), 805 (0.0, Au), 747 (0.0, Bu), 728 (55, Ag, ν_sBrO_2), 706 (0.0, Bu, ν_sBrO_2), 700 (5.6, Ag), 278 (42, Ag), 264(0.0, Bu), 218 (0.0,m Au), 213 (0.0, Bu), and five vibrations below 200 cm^{-1}.

References

[S1] SHELXS, program for crystal structure solution, G. M. Sheldrick, 1986 – 2013, version 2013/1; SHELXL, program for crystal structure refinement, G. M. Sheldrick, program for crystal structure refinement 1993 – 2014, version 2014/7.

[S2] Gaussian 16, Revision A.03, M. J. Frisch, G. W. Trucks, H. B. Schlegel, G. E. Scuseria, M. A. Robb, J. R. Cheeseman, G. Scalmani, V. Barone, G. A. Petersson, H. Nakatsuji, X. Li, M. Caricato, A. V. Marenich, J. Bloino, B. G. Janesko, R. Gomperts, B. Mennucci, H. P. Hratchian, J. V. Ortiz, A. F. Izmaylov, J. L. Sonnenberg, D. Williams-Young, F. Ding, F. Lipparini, F. Egidi, J. Goings, B. Peng, A. Petrone,T. Henderson, D. Ranasinghe, V. G. Zakrzewski, J. Gao, N. Rega, G. Zheng, W. Liang, M. Hada, M. Ehara, K. Toyota, R. Fukuda, J. Hasegawa, M. Ishida, T. Nakajima, Y. Honda, O. Kitao, H. Nakai, T. Vreven, K. Throssell, J. A. Montgomery, Jr., J. E. Peralta,F. Ogliaro, M. J. Bearpark, J. J. Heyd, E. N. Brothers, K. N. Kudin, V. N. Staroverov, T. A. Keith, R. Kobayashi, J. Normand, K. Raghavachari, A. P. Rendell, J. C. Burant, S. S. Iyengar, J. Tomasi, M. Cossi, J. M. Millam, M. Klene, C. Adamo, R. Cammi, J. W. Ochterski, R. L. Martin, K. Morokuma, O. Farkas, J. B. Foresman, and D. J. Fox, Gaussian, Inc., Wallingford CT, 2016.

[S3] J. F. Lehmann, S. Riedel, G. J. Schrobilgen, *Inorg. Chem*. **2008**, *47*, 8343 – 8356.

[S4] Supplementary crystallographic data can be obtained free of charge from the Cambridge Crystallographic Data Centre by quoting CCDC 1953518 $(BrOF_2)_3$*(acetone)$_4$, CCDC 1952910 ($BrO_2^+AsF_6^-$), CCDC 1953515 ($BrO_2^+AsF_6^-$*$2BrO_2F$), CCDC 1953432 ($Br_3O_4^+$ *Br_2 AsF_6^-), CCDC 1953181 (BrO_2^+ $H(OSO_2CF_3)_2^-$), CCDC 1953176 ($Br_3O_6^+$ $OSO_2CF_3^-$), CCDC 1953428 ($Cl_2BrO_6^+$ $Mo_3O_3F_{13}^-$), CCDC 1953427 ($Cl_2BrO_6^+$ ClO_4^- $HF_{0.5}$), CCDC 1958076 (O_2Br-O-$COCF_3$), and CCDC 1953431 ($NO_2^+Br(ONO_2)_2^-$).

[S5] A. K. Wilson, D. E. Woon, K. A. Peterson, T. H. Dunning jr., *J. Chem. Phys*. **1999**, *110*, 7667 – 7676.
